# Biallelic Mutations in *ACACA* Cause a Disruption in Lipid Homeostasis That Is Associated With Global Developmental Delay, Microcephaly, and Dysmorphic Facial Features

**DOI:** 10.3389/fcell.2021.618492

**Published:** 2021-09-06

**Authors:** Xiaoting Lou, Xiyue Zhou, Haiyan Li, Xiangpeng Lu, Xinzhu Bao, Kaiqiang Yang, Xin Liao, Hanxiao Chen, Hezhi Fang, Yanling Yang, Jianxin Lyu, Hong Zheng

**Affiliations:** ^1^Key Laboratory of Laboratory Medicine, Ministry of Education, Zhejiang Provincial Key Laboratory of Medical Genetics, College of Laboratory Medicine and Life Sciences, Wenzhou Medical University, Wenzhou, China; ^2^The First Affiliated Hospital of Henan University of Traditional Chinese Medicine, Zhengzhou, China; ^3^Department of Pediatrics, Peking University First Hospital, Beijing, China; ^4^Zhejiang Provincial People’s Hospital, Affiliated People’s Hospital of Hangzhou Medical College, Hangzhou, China

**Keywords:** lipogenesis, palmitate, acetyl-CoA carboxylase 1, developmental delay (DD), *ACACA* gene

## Abstract

**Objective:**

We proposed that the deficit of ACC1 is the cause of patient symptoms including global developmental delay, microcephaly, hypotonia, and dysmorphic facial features. We evaluated the possible disease-causing role of the *ACACA* gene in developmental delay and investigated the pathogenesis of ACC1 deficiency.

**Methods:**

A patient who presented with global developmental delay with unknown cause was recruited. Detailed medical records were collected and reviewed. Whole exome sequencing found two variants of *ACACA* with unknown significance. ACC1 mRNA expression level, protein expression level, and enzyme activity level were detected in patient-derived cells. Lipidomic analysis, and *in vitro* functional studies including cell proliferation, apoptosis, and the migratory ability of patient-derived cells were evaluated to investigate the possible pathogenic mechanism of ACC1 deficiency. RNAi-induced ACC1 deficiency fibroblasts were established to assess the causative role of ACC1 deficit in cell migratory disability in patient-derived cells. Palmitate supplementation assays were performed to assess the effect of palmitic acid on ACC1 deficiency-induced cell motility deficit.

**Results:**

The patient presented with global developmental delay, microcephaly, hypotonia, and dysmorphic facial features. A decreased level of ACC1 and ACC1 enzyme activity were detected in patient-derived lymphocytes. Lipidomic profiles revealed a disruption in the lipid homeostasis of the patient-derived cell lines. *In vitro* functional studies revealed a deficit of cell motility in patient-derived cells and the phenotype was further recapitulated in ACC1-knockdown (KD) fibroblasts. The cell motility deficit in both patient-derived cells and ACC1-KD were attenuated by palmitate.

**Conclusion:**

We report an individual with biallelic mutations in *ACACA*, presenting global development delay. *In vitro* studies revealed a disruption of lipid homeostasis in patient-derived lymphocytes, further inducing the deficit of cell motility capacity and that the deficiency could be partly attenuated by palmitate.

## Introduction

Acetyl-coenzyme A carboxylase (ACC) catalyzes the conversion of acetyl-CoA into malonyl-CoA (Hopwood and Sherman, 1990). In mammalian cells, two tissue-specific isoforms (ACC1 and ACC2) of ACC exist. Cytosolic protein ACC1 mainly exists in the liver and adipose tissue (Thampy, 1989) and is encoded by ACC alpha (*ACACA*, MIM: 200350). In contrast, mitochondrial membrane protein ACC2 mainly exists in the liver, heart, and skeletal muscle (Abu-Elheiga et al., 2000) and is encoded by ACC beta (*ACACB*, MIM:601557). ACC1 serves as the first and rate-limiting enzyme in *de novo* fatty acid biosynthesis. Malonyl-CoA produced by ACC1, serves as the substrate not only for *de novo* lipogenesis but also for further fatty acid chain elongation (Wakil and Abu-Elheiga, 2009). The malonyl-CoA produced by ACC2 works as the inhibitor of carnitine palmitoyltransferase 1 (CPT1), and participates in the regulation of fatty acid oxidation (Abu-Elheiga et al., 2001). Collectively, both ACCs play essential roles in lipid metabolism.

Studies revealed that the lack of ACC1 would lead to an alteration in the lipid composition (Glatzel et al., 2018), as malonyl-CoA is the substrate for fatty acid chain elongation. The homeostasis of lipid composition is required for several cell metabolism processes including cell proliferation, cell death (Agmon and Stockwell, 2017), and cell motility (Glatzel et al., 2018). Numerous of studies have focused on revealing the role ACC1 plays in metabolic and neoplastic diseases (Milgraum et al., 1997; Harwood, 2004, 2005; Berod et al., 2014; Rios Garcia et al., 2017; Stiede et al., 2017; Glatzel et al., 2018). Despite this, the implication of *ACACA* in Mendelian diseases has yet to be elucidated. The first patient with ACC deficiency was described in Blom et al. (1981). Low ACC enzyme activity was detected in a liver biopsy while direct sequencing evidence was lacking due to the limitation of technology; it was unable to tell whether the ACC deficiency was caused by the mutations in *ACACA* or *ACACB*.

Here, we first describe a patient with *ACACA* mutations with the support of direct sequencing evidence. Functional assays were performed to investigate the pathogenesis of ACC1 deficiency and possible therapeutic chemical input. However, further conclusions about genotype-phenotype correlations require more clues in the future and should be carefully characterized at a clinical level.

## Materials and Methods

### Study Participants

The individual was first evaluated at the Pediatric Clinic at Peking University First Hospital and the follow-up evaluations were performed at the Pediatric Clinic at The First Affiliated Hospital of Henan University of Chinese Medicine. Genetic testing was performed at the Chi-gene (China). The proband provided peripheral blood for further molecular analysis. All family members provided written informed consent to participate in this study. This study was approved by the Ethics Committee of Peking University First Hospital (approval study ID: 2017-217).

### Variants Analysis

Whole exome sequencing (WES) was performed using an Illumina HiSeq 2000 sequencer (Illumina, United States). The bioinformatic work followed sequencing. Briefly, the bioinformatic workflow pipeline included quality control of raw data, processing data, aligning data to reference genome (GRCh38/hg38), variant calling, genome assembly, and genome annotation. Furthermore, the candidate disease-causing variants were filtered with the following criteria: Data quality (most probable genotype, MPG score > 10; Sims et al., 2014); compound heterozygous or homozygous variants; non-synonymous/non-sense/splice/frameshift variants; variants that were absent in the dbSNP database, 1,000 genomes or controls. DNA were extracted from all three participants (proband, father, and mother) as described before (Lou et al., 2018), and Sanger sequencing was performed using specific primers (Supplementary Table 2) for segregation analysis.

### Bioinformatic Analysis

The possible effect caused by both variants was predicted by using the following online bioinformatic analysis tools: SIFT^1^ (Vaser et al., 2016), PROVEAN^2^ (Choi and Chan, 2015), MutationTaster^3^ (Schwarz et al., 2014), PolyPhen-2^4^ (Adzhubei et al., 2010), dbSNP^5^ (Sherry et al., 2001), ClinVar^6^ (Landrum et al., 2020), ExAC^7^, and SDM^8^ (Worth et al., 2011; Pandurangan and Blundell, 2020).

### Immortalization of B Lymphocytes

Peripheral blood mononuclear cells (PBMCs) were extracted from the individual and age-matched controls’ whole blood. Immortalization of B lymphocytes followed the procedure described before (Lou et al., 2018). Immortalized lymphoblastoid cells were cultured with Roswell Park Memorial Institute (RPMI) 1,640 medium (Thermo Fisher Scientific, United States) supplemented with 10% Fetal Bovine Serum (FBS; Gibco, United States), 1% (v/v) penicillin-streptomycin (Beyotime Biotechnology), and 2.5 μg/mL of amphotericin (Sangon, China) at 37°C and 5% CO_2_.

### Cell Culture and Construction of RNAi ACC1-Knockdown Cell Lines

Fibroblasts were obtained from healthy children’s foreskin following the protocol approved by The First Affiliated Hospital of Wenzhou Medical University. The human umbilical vein endothelial cells (HUVEC) cell line was a gift from Dr. Licai He’s lab. ACC1 was knocked down in both fibroblasts and the HUVEC cell line by RNAi duplex constructs (Supplementary Table 2) using lipofectamine RNAiMAX (Thermo Fisher Scientific) according to the manufacturer’s instructions. All fibroblasts were cultured in Dulbecoo’s Modified Eagle Medium (DMEM; Gibco) supplemented with 10% FBS (Gibco), 1% (v/v) penicillin-streptomycin (Beyotime Biotechnology), and 2.5 μg/mL of amphotericin (Sangon). The HUVEC cell line was cultured in Gibco RPMI 1,640 medium containing 10% FBS (Gibco), 1% (v/v) penicillin-streptomycin (Beyotime Biotechnology), 2.5 μg/mL of amphotericin (Sangon), and 25 mM of HEPES (Solarbio, China). All cells were cultured at 37°C and 5% CO_2_.

### Real-Time PCR

Real-Time PCR was performed as described previously (Fang et al., 2018). Briefly, total RNA was extracted from lymphocytes or fibroblasts and reverse-transcribed into cDNA using TRIzol^TM^ Reagent (Thermo Fisher Scientific) or a PrimeScript^TM^ RT Master Mix (Perfect Real Time; Takara Biotechnology, China), respectively, according to the manufacturer’s instructions. The RT-PCR reaction was performed using the Universal SYBR Green Supermix (Bio-Rad, United States) according to the manufacturer’s instructions. All primers are listed in Supplementary Table 2.

### Cytoplasm Extraction

Considering that both ACC1 and ACC2 have ACC activity, cytoplasm was extracted to avoid the interference from ACC2. Cytoplasm was extracted as described before (Bai et al., 2017). Briefly, either 1 × 10^6^ lymphocytes or fibroblasts were harvested and washed with ice-cold phosphate-buffered saline (PBS), cells were incubated with 100 μl of ice-chilled extracting buffer [150 mM of NaCl (Sigma-Aldrich, United States), 50 mM of HEPES (Sigma-Aldrich, pH 7.4), and 12.5 μg/mL OF digitonin (Sigma-Aldrich)] for 1 min and subjected to centrifugation at 980 × *g* for 5 min, followed by additional centrifugation twice at 980 × *g* for 5 min. Cytoplasm was obtained by a final centrifuge of the supernatant at 20,000 × *g* for 25 min (Bai et al., 2017). The cytoplasm sample was finally divided evenly into two parts: One for ACC1 protein detection by SDS-PAGE, and one for ACC1 enzymatic activity measurement.

### SDS-PAGE

For lymphocytes, cytoplasm samples outlined above for SDS-PAGE were used to detect ACC1 protein levels. For fibroblasts, whole cell lysis applied for SDS-PAGE was used to detect ACC1 protein levels. Fibroblasts were harvested and lysed in RIPA lysis buffer (Cell Signaling Technology, United States) with protease inhibitor (Beyotime Biotechnology, China); cytoplasm sample: Outlined above. Protein concentration was determined using a Pierce^TM^ BCA Protein Assay Kit (Thermo Fisher Scientific) following the manufacture’s instructions. Briefly, BCA working reagent was prepared by mixing 50 parts of BCA reagent A with 1 part of BCA reagent B. A total of 1 μl of sample was pipetted into the 96-well plate containing 0.2 ml of working reagent, mixed well, and incubated at 37°C for 30 min. The absorbance of all samples was measured with the spectrophotometer set to 562 nm. The protein concentration was quantified by the standard curve of a set of protein standards. The denaturation of the protein was performed at 95°C for 5 min. Later, SDS-PAGE was performed as described before (Lou et al., 2018). Overall, 30 μg of total protein was loaded into 8% polyacrylamide gels, then the protein was transferred onto polyvinylidene difluoride membranes (PVDF; Bio-Rad). The membrane was blocked with 5% no-fat milk for 2 h and probed with appropriate primary antibodies overnight: Anti-ACC1 (1:1,000, Proteintech, China) and anti-β-actin (1:2,000, Abcam, United Kingdom). Horseradish peroxidase marked anti-mouse antibody or anti-rabbit antibody (1:2,000, Cell Signaling Technology) was used. Protein signal was detected by western ECL substrate (Bio-Rad).

### ACC1 Enzyme Activity Measurement

Cytoplasm samples outlined above were used for further ACC1 enzyme activity measurement. ACC1, rather than ACC2, was measured based on a discontinuous spectrophotometric method. Enzyme activity of ACC1 was determined as follows (Willis et al., 2008; Sumiya et al., 2015): 10 μl of ACC1-containing cytoplasm was added into pre-warmed (30°C) ACC reaction buffer [100 mM of potassium phosphate (Sigma-Aldrich, pH 8.0), 15 mM of KHCO3 (Sangon), 5 mM of MnCl2 (Sigma-Aldrich), 1 mg/mL of bovine serum albumin (BSA, Beyotime Biotechnology, China), 1 mM of acetyl-CoA (Sigma-Aldrich), 5 mM of ATP (Sigma-Aldrich), and 3 mg/L of biotin (Sigma-Aldrich)] to start the conversion of acetyl-CoA to malonyl-CoA in 30°C for 15 min. The untransformed acetyl-CoA in ACC reaction buffer was determined by incubating the cytoplasm-containing ACC reaction buffer with acetyl-CoA assay buffer [100 mM of potassium phosphate (pH 8.0), 0.1 mg/mL of dithionitrobenzoic acid (DTNB, Sigma-Aldrich), 20 mM of oxaloacetate (Sigma-Aldrich), and 1 mg/mL of BSA, 0.5 unit citrate synthase (Sigma-Aldrich)] at 30°C until the absorbance at 412 nm was not changed. The relative ACC1 activity normalized to overall cytoplasm protein was calculated by [OD412 (ACC reaction buffer)-OD412 (ACC reaction buffer with cytoplasm proteins)]/15 min/μg of cytoplasm protein.

### Cell Preparation and Lipidomic Analysis

Lipidomics was performed by the Novogene company (China). Lymphocytes were harvested and rapidly frozen by liquid nitrogen. Lipid extraction, UHPLC-MS/MS analysis, and a data search were performed successively. Finally, heat maps were clustered and the correlation between differential metabolites was analyzed. *P*-value <0.05 was considered as statistically significant.

### Trans-Well Migration Assay

The Trans-well migration assay was performed as described before (Glatzel et al., 2018). The lymphocytes (1 × 10^5^) were added into the upper chamber (5 μm) containing RPMI 1640 (Thermo Fisher Scientific) with 0.1% FBS (Gibco) and the lower chamber with 20% FBS (Gibco). The cell numbers of both the upper and the lower chamber were counted after 6 h using a flow cytometer. The fibroblasts (1 × 10^5^) were seeded into the upper chamber (8 μm) containing DMEM with 0.1% FBS (Gibco) and the lower chamber with 20% FBS (Gibco). After 24 h, the cells were fixed with 4% paraformaldehyde (Lingfeng, China), stained with crystal violet (Beyotime Biotechnology), and then observed by microscope (Nikon, Japan). Lymphocytes were pre-treated with PA (150 μM, Sigma-Aldrich) dissolved in 20% fatty acid-free bovine serum albumin (Solarbio, China) for 24 h and then migrated for 6 h. The PA (50 μM) was added into both upper and lower chambers during the migration of fibroblasts, and the remaining steps were performed as before.

### Wound Healing Assay

The wound healing assay was performed as described (Sims et al., 2014). Briefly, fibroblasts (1 × 10^5^) were seeded into a 6-well plate in culture medium with or without PA (50 μM). After 24 h, a line was scratched by a sterile pipette tip into cell monolayers, and then washed with pre-warmed PBS twice. The scratch wound was allowed to recover for 24 h, micrographs were captured for each sample at the same time points (0, 12, and 24 h) using a microscope (Nikon, Japan). Image J software (NIH, United States) was used to quantify the area of the scratch of each sample. The wound healing assay of HUVEC cell lines was performed as for fibroblasts. The HUVEC cell lines (5 × 104) was seeded into a 6-well plate in culture medium with or without PA (50 μM). After scratching, the cell was allowed to cover for 12 h, and micrographs were captured for each sample at the same time points (0, 6, and 12 h).

### Apoptosis Analysis

The cellular apoptosis assay was performed using FITC Annexin V Apoptosis Detection Kit I (Biosciences, United States) according to the manufacturer’s instructions. In short, the cells were stained with FITC and PI for 15 min at room temperature in the dark. Finally, fluorescence of FITC annexin V and PI were detected by a flow cytometer.

### Cell Proliferation Assay

Lymphocytes (1 × 10^5^) were seeded into a 6-well plate with culture medium, and the cell number was determined by a flow cytometer at the same time points (0, 24, 48, and 72 h).

### Statistical Analyses

All experiments were performed in triplicate and independently at least three times. All data were analyzed by mean ± SEM in prism 8.0 (GraphPad, United States). *P*-values were calculated using independent Student’s *t* test or one-way *ANOVA*, *p* < 0.05 was considered statistically significant.

## Results

### Clinical Information

The proband (II-1) was born to a non-consanguineous Chinese family. The patient was evaluated at 17 months at the local hospital with the diagnosis of motor developmental delay, intellectual developmental delay, hypotonia, and elevated lactate (3.4 mM, normal 0–2.2 mM). Then the patient was referred to the Pediatric Clinic at Peking University First Hospital at 25-months-old for diagnosis of global developmental delay. The birth history was obtained from the parents, the proband was born after a 40-week gestation via cesarean section due to premature rupture of membranes (PROM) and amniotic fluid contamination. Her birth weight was 3.3 kg, birth length was 50 cm. A detailed physical examination was done at 25 months: Body height: 83 cm (−2SD <<−1SD), body weight: 11.5 kg (−2SD <<−1SD), head circumference: 43.5 cm (<−3SD), no abnormality in vision and hearing, no rashes in skin/breast, normal bowel sounds, abdomen soft, and non-tender. Laboratory investigations revealed an elevated blood lactate level (2.69, 0.7–2.1 mM), hypoxemia (10.7, 15–22 ml/dl), an elevated urine amino acid level (Arg/Orn 0.01, normal 0.03–0.70; Cit/Arg 11.15, normal 0.33–8.01), and a decreased or low limit of the normal region in the serum carnitine assay (C4 0.1 μM, normal 0.15–1.85 μM; C5 0.01 μM, normal 0.01–0.14 μM; C6 0.01 μM, normal 0.01–0.14 μM; C16 0.53 μM, normal 0.75–6.2 μM, and C5/C8 0.17, normal 0.19–7.00). There were unremarkable changes in the urine organic acid assay and blood ceruloplasmin level. Magnetic resonance imaging (MRI) showed that the sulcus and fissures of bilateral cerebral area were deepening as indicated (Supplementary Figure 1A), which mostly correlated to the malformation of the brain and a deficit in cognitive ability. Unremarkable abnormalities were apparent in the electrocardiogram (EEG) and echocardiogram (ECG). Other remarkable medical records including fatigue, muscle weakness, and language disorder were noted. Genetic testing revealed compound heterozygous variants in the *ACACA* gene. Vitamin B2 was applied with the consideration of the symptoms of weakness, fatigue, etc., Levocarnitine was administered to attenuate the deficiency of serum carnitine. As ACC1 is the key limited enzyme during the fatty acid synthesis process, medium-chain triglycerides (MCT) oil was supplemented to compensate the deficit of fatty acid synthesis. Concisely, the following medication was applied at 25 months: Vitamin B2 (60 mg/d), levocarnitine (0.5 g/d), and MCT oil (10 ml/d). The individual was evaluated with the Griffith Mental Development Scales (GMDS) according to the instructions of the follow-ups (detailed in Supplementary Table 1). The first evaluation was performed at 25 months (before treatment). The second evaluation was done after 19 months of treatment. Compared to the first evaluation, personal social emotional (36–41%, equivalent age/actual age), language and communication (30–45%), eye and hand coordination (20–41%), and the vision of the patient (34–49%) had improved by the second evaluation, with no improvement in the gross motor quotient (both 44%). At 51 months (26 months after treatment), similar to the pattern at 44 months, her language and communication (30–48%), eye and hand coordination (20–37%), and vision displayed improvements (34–46%). According to the parents, they got the feeling that the patient also improved in exercise endurance and language ability after treatment. However, due to the limitation of the patient number, all the evaluations failed to exclude the normal biological development effect.

### Pathogenicity Evaluation of *ACACA* Variants

**FIGURE 1:**
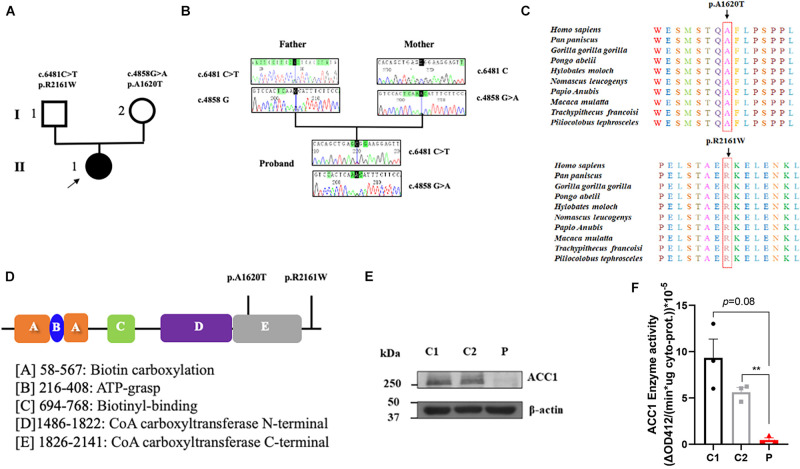
Summary of the genetic characteristics and SDS-PAGE of ACC1 protein content and ACC1 enzyme activity. **(A)** The pedigree of the affected individual. The proband was represented as black solid circle and pointed out by an arrow. The square indicates male, and the circle female. **(B)** Sanger sequencing of the mutations. **(C)** The amino acid conservation among different species. **(D)** The functional domain of ACC1, and the mutation sites were marked. **(E)** Relative ACC1 protein content of lymphocytes. **(F)** The ACC1 enzyme activity among the proband- and age-matched control-derived lymphocytes. One-way *ANOVA* applied in panels **(E,F)**. **p < 0.01.

To investigate the genetic basis of the patient (II-1), WES was performed. According to the criterion mentioned earlier, two compound heterozygous missense variants in *ACACA* (NM_198839), c.4858G > A (p. Ala1680Thr) and c.6481C > T (p. Arg2161Trp) were harbored in the patient (Figure 1A). All variants were confirmed by Sanger sequencing. Segregation analysis revealed that the patient’s mother carries c.4858G > A and father carries the c.6481C > T (Figure 1B). p.A1620 and p.R2161 are both highly conserved among species (Figure 1C). The American College of Medical Genetics and Genomics (ACMG) guideline (Richards et al., 2015) for variants classification revealed that c.4858G > A was “pathogenic,” and c.6481C > T was “likely pathogenic.” Both mutations were absent from public databases (including dbSNP, 1,000 Genome, ClinVar, and gnomAD; Table 1). Prediction software including SIFT, MutationTaster, and PolyPhen-2 were used to predict the possibility of the amino acid substitution effect on protein function. Accordingly, details are presented in Table 1. The mutation sites are located in CoA carboxyltransferase C-terminal and in the functional domain, respectively (Figure 1D). An *in silico* model was applied to predict the effect of amino acid substitution. Site Directed Mutator (SDM)-predicted mutation induced changes in protein thermodynamic stability in Gibbs; both Ala1680Thr and Arg2161Trp have negative effects on the protein stability, especially the Ala1680Thr (Table 2).

**TABLE 1:** Bioinformatic analysis of *ACACA* mutations.

Nucleotide change	c.4858G > A	c.6481C > T
Amino acid change	p. A1620T	p. R2161W
SIFT^1^	Tolerated (score = 0.515)	Tolerated (score = 0.105)
PROVEAN^2^	Neutral (score = −0.65)	Deleterious (score = −5.778)
MutationTaster	Disease-causing	Disease-causing
PolyPhen-2^3^	Benign (score = 0.014)	Probably damaging (score = 0.999)
dbSNP (MAF/minor allele count)^4^	−(<0.005)	−(<0.005)
ClinVar^5^	–	–
ExAC allele frequency^6^	0.0001	0.0009

*^1^The SIFT score ranges from 0.0 (deleterious) to 1.0 (tolerated); variants with scores closer to 0.0 are more confidently predicted to be deleterious, and very close to 1.0 are more confidently predicted to be tolerated.*

*^2^The cutoff for PROVEAN scores was set to -2.5. That is, consider a score higher than –2.5 to be neutral (tolerated) and that lower than or equal to –2.5 to be deleterious (damaging).*

*^3^The PolyPhen-2 score ranges from 0.0 (tolerated) to 1.0 (deleterious). Between 0.0 and 0.15 – variants with scores in this range are predicted to be benign; 0.15 and 1.0 – variants with scores in this range are possibly damaging; 0.85 and 1.0 – variants with scores in this range are more confidently predicted to be damaging.*

*^4^The “–” means both variants were absent from dbSNP.*

*^5^The “–” means no data have been submitted to the ClinVar database.*

*^6^The ExAC allele frequency represents the heterozygous mutation frequency.*

**TABLE 2:** SDM* prediction of *ACACA* mutations.

Mutation	PDB file**	Chain ID	Predicted ΔΔG***	Outcome
A1620T	6G2D.pdb	D	−1.55	Reduced stability
R2161W	6G2D.pdb	C	−0.3	Reduced stability

*SDM, site directed mutator is a computational method used to analyze the effects of mutations on protein stability. The results are displayed with predicted pseudo ΔΔG, a negative value corresponds to a mutation predicted to be destabilizing, accordingly, a positive value corresponds to a stabilizing mutation.

**The 3D structures are both available in the Protein Data Bank (PDB).

***The results displayed with predicted pseudo ΔΔG, a negative value corresponds to mutation predicted to be destabilizing, accordingly, a positive value corresponds to a stabilizing mutation.

### Identification of ACC1 Deficiency in Patient-Derived Lymphocytes

In order to investigate the effect of the compound heterozygous variants on ACC1 expression, RT-PCR and western blot were used to detect the ACC1 expression levels in patient-derived lymphocytes. The mRNA levels of ACC1 showed no significant decrease compared to the age-matched healthy controls (Supplementary Figure 1B), while the protein levels of ACC1 in the patient were decreased remarkably compared to controls (Figure 1E). The ACC1 enzyme activity assay unveiled that ACC1 enzyme activity in patient-derived cells was decreased 95% (*p* = 0.08) and 90% (*p* = 0.007) compared to C1 and C2, when normalized to the overall cytoplasm protein level (Figure 1F). Furthermore, the relative ACC1 enzyme activity in patient-derived cells was decreased 70% (*p* = 0.12) and 60% (*p* = 0.025) compared to C1 and C2, when normalized to the relative corresponding ACC1 protein levels in cytoplasm (Supplementary Figures 1C,D). However, considering that western blot is a semi-quantification method and may not be reliable for absolute quantification of ACC1, we should be very careful to draw the conclusion that the activity was affected by the mutations.

### Disruption of Lipid Homeostasis in Patient-Derived Lymphocytes

**FIGURE 2:**
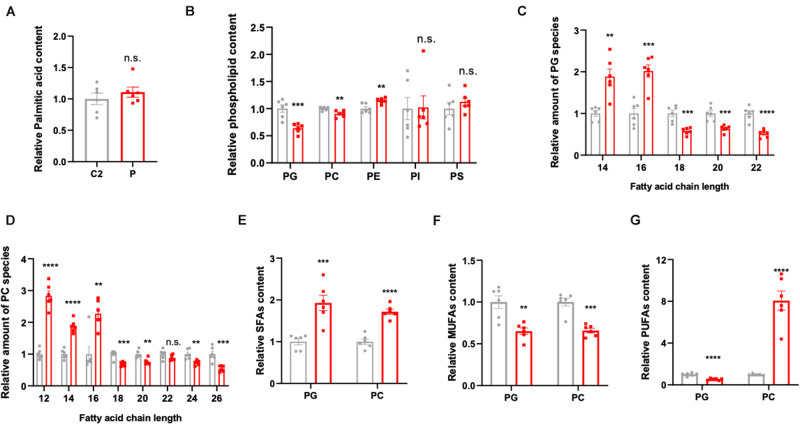
Lipidomics analysis in the patient- and control-derived lymphocytes. **(A)** Relative palmitic acid content in the lymphocytes. **(B)** The quantification of membrane lipid components including phosphatidylglycerol (PG), phosphatidylcholine (PC), phosphatidylethanolamine (PE), phosphatidylinositol (PI), and phosphatidylserine (PS). Relative amount of substance in **(C)** PG and **(D)** PC, analyzed based on fatty acid chain length. **(E)** The saturated fatty acids (SFAs), **(F)** monounsaturated fatty acids (MUFAs), and **(G)** polyunsaturated fatty acids (PUFAs) were analyzed in PG and PC, respectively. All data were from six independent replicates and analyzed by mean ± SEM. Independent *t*-test in panels **(A–G)**. **p < 0.01, ***p < 0.001, ****p < 0.0001.

In order to explore the pathogenesis of ACC1 deficiency, patient- and age-matched control-derived lymphocytes were used to perform lipidomic profile analysis. The results indicate a disruption of lipid homeostasis in the patient-derived lymphocytes compared to the age-matched control. As palmitic acid (16:0) is the first product in *de novo* lipogenesis, palmitic acid levels were analyzed; however, there was no significant changes in the palmitic acid levels of the patient-derived lymphocytes (Figure 2A). The lipid composition shifted in patient cells compared to control’s, the amounts of PG, PC, and PE were decreased in the patient while there were no significant changes in the content of PI and PS (Figure 2B). Considering that malonyl-CoA is produced by ACC1, it will also participate in the elongation of fatty acid chains besides the *de novo* lipogenesis. Considering PG and PC are the main membrane components, further analysis according to different chain lengths was conducted. Interestingly, the longer chain (*n* > 16) fatty acids were reduced in the patient-derived lymphocytes in both PG and PC (Figures 2C,D), indicating that the lack of ACC1 affects the elongation of fatty acid chain. Furthermore, the levels of saturated fatty acids (SFAs), monounsaturated fatty acids (MUFAs), and polyunsaturated fatty acids (PUFAs) were also analyzed. As is shown in Figures 2E–G, the level of SFAs was increased in the patient-derived lymphocytes; however, MUFAs were decreased. The PUFAs of the patient were decreased in PG but increased in PC. In summary, ACC1 deficiency mainly leads to a shift in membrane lipid composition, and the longer chain fatty acids (*n* > 16) in the membrane lipid composition, such as phosphatidylglycerol (PG) and phosphatidylcholine (PC), declined.

### The Cell Migratory Ability Was Deficient in Patient-Derived Lymphocytes and Partly Attenuated by Palmitate

**FIGURE 3:**
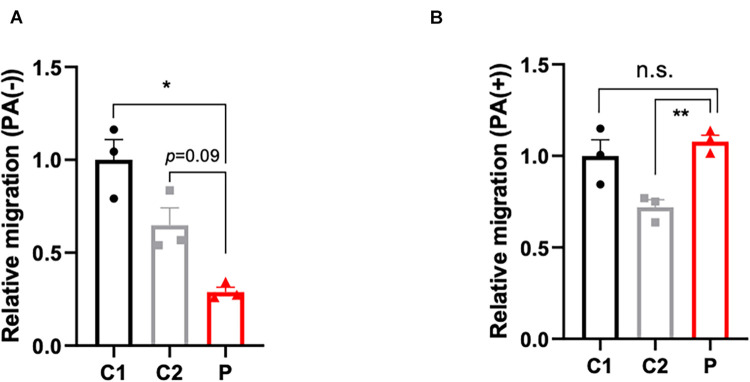
Migration capacity of lymphocytes with or without palmitate supplement. **(A)** Relative migration of patient- and control-derived lymphocytes without palmitate supplement and **(B)** with palmitate. One-way *ANOVA* applied in panels **(A,B)**. *p < 0.05, **p < 0.001.

The disturbance of lipid metabolism impacts cell apoptosis (Pohle et al., 2004), cell proliferation (Zhu and Thompson, 2019), and cell motility. In order to ascertain the impact of lymphocytes lipid disturbance, apoptosis (Supplementary Figures 2A–C) and proliferation assays (Supplementary Figure 2D) were conducted, however, no significant change between patient- and control-derived lymphocytes was found. Nevertheless, the cell motility capacity was remarkably decreased in the patient-derived lymphocytes (Figure 3A). Considering that malonyl-CoA would participate in not only the production of palmitic acid, but also the elongation of fatty acids, palmitate was supplemented to compensate for the deficit of malonyl-CoA caused by ACC1 deficiency. As expected, the migration ability in the patient-derived lymphocytes improved when treated with palmitate (Figure 3B).

### ACC1-KD Fibroblast Cell Lines Were Deficient in Cell Motility and Partly Attenuated by Palmitate

**FIGURE 4:**
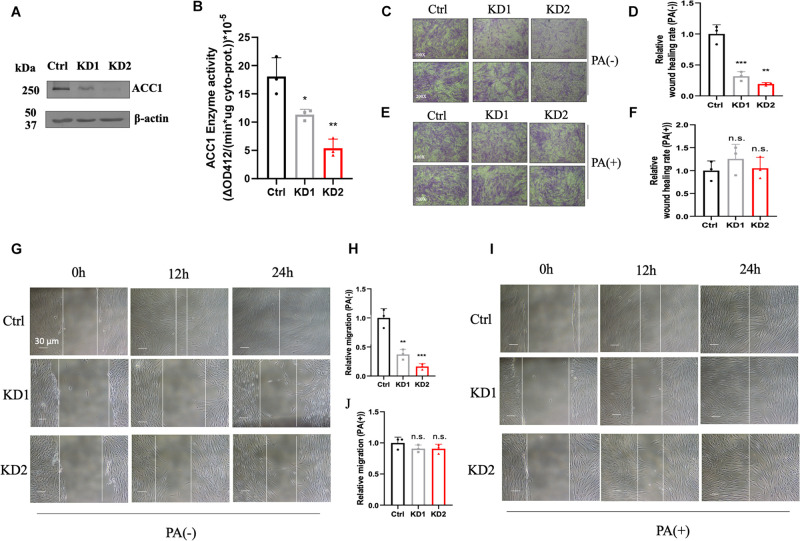
ACC1-knockdown (KD) fibroblasts and the cell motility capacity measurement. **(A)** The ACC1 protein content of ACC1-KD fibroblast cell lines, and **(B)** the ACC1 enzyme activity. The wound healing assay was carried out at different time points (0, 12, and 24 h) **(C)** without or **(E)** with palmitate (scale bar = 30 μm), and **(D,F)** show quantitative analysis, respectively. The Trans-well migration capacity at 100× magnification (upper) and 200× magnification (lower) **(G)** with or **(I)** without palmitate, respectively. **(H,J)** The quantitative analyses, respectively. All data were from three independent replicates and analyzed by mean ± SEM. Independent *t*-test in panels **(A,B,D,F,H,J)**. *p < 0.05, **p < 0.01, ***p < 0.001.

In order to investigate the effect of lipid disorder, ACC1-KD fibroblast cell lines were conducted by RNAi duplex constructs. The mRNA levels in KD1 and KD2 were decreased by about 69 and 75% compared to the wild-type fibroblasts (Supplementary Figure 3A) and protein content were both decreased in two ACC1-KD cell lines (Figure 4A). The enzyme activities of ACC1 were decreased by 37 and 70%, respectively (Figure 4B). The wound healing assay and the Trans-well migration assay were conducted in fibroblasts for migration capacity detection. The wound healing capacity was suppressed in the ACC1-KD fibroblasts compared to the wild-type cells (Figures 4C,D). The migration ability was also suppressed (Figures 4G,H). The wound healing capacity (Figures 4E,F) and the migration ability (Figures 4I,J) of ACC1-deficient fibroblast cell lines were both improved with palmitate supplement.

## Discussion

Fatty acid metabolism includes fatty acid synthesis and fatty acid oxidation. ACC catalyzes the conversion of acetyl-CoA into malonyl-CoA (Wakil and Abu-Elheiga, 2009). Malonyl-CoA further participates in *de novo* fatty acid biosynthesis. Fatty acid oxidation disorders (FAODs) were first described in the 1970s, and from then on numerous FAODs have been reported (Merritt et al., 2018). FAODs are a group of genetic clinical heterogeneous rare disorders caused by the disruption of mitochondrial β-oxidation or the fatty acid carnitine transport pathway (Deschauer et al., 2005; Clemente et al., 2014; Miller et al., 2015). FAODs mostly present with severe cardiomyopathy, hypoketotic hypoglycemia, liver dysfunction, and episodic rhabdomyolysis (Merritt et al., 2018). However, an association between lipid biosynthesis and Mendelian diseases have not yet been revealed. A possible ACC deficiency patient was described in 1981, giving evidence of low ACC enzyme activity (Blom et al., 1981). Due to the limitation of sequencing technology, it was difficult to tell the causation (*ACACA* or *ACACB*) of enzyme deficiency.

We described a patient presenting with global developmental delay harboring bi-allelic mutations in *ACACA* (c.4858G > A [p. Ala1680Thr] was inherited maternally, and c.6481C > T [Arg2161Trp] was inherited paternally). Both mutations are missense mutations causing only a single amino acid change in each chain. Both p. Ala1680 and p. Arg2161 are highly conserved across the species. The mutation of p. Ala1680Thr was located in the CoA carboxyltransferase C-terminal. Evidence provided by bioinformatic tools including *in silico* and 3D protein modeling prediction models corroborated the pathogenicity of the mutations. RT-PCR was firstly performed to evaluate the ACC1 transcription of patient-derived cells, and revealed that there was no significant effect on the ACC1 transcription. Western blot suggested that translation in patient-derived cells was affected. Considering ACC1 is a key enzyme in lipogenesis, enzymatic activity was measured. ACC1 enzyme activity in patient-derived cells was almost 10 and 5% of that in C1 and C2 when normalized to cytoplasm protein amount. Furthermore, in order to investigate the possible effect of mutations on ACC1 enzymatic activity besides ACC1 transcription, ACC1 enzyme activity was further normalized with relative cytoplasm-ACC1 protein amount after firstly being normalized to total cytoplasm protein. The ACC1 enzyme activity per unit in patient-derived cells was almost 30 and 40% per unit in C1 and C2 (Supplementary Figure 1C). The combined results indicate that compound heterozygous mutations may not only affect the transcription process but also residue kinetics without affection to the translation. However, considering that western blot is a semi-quantification method and may not be reliable for absolute quantification of ACC1, we should be very careful to draw the conclusion that the activity was affected by the mutations. The results of ACC1 enzymatic activity coincided with SDM prediction. SDM (Pandurangan and Blundell, 2020) is a computational method that analyzes the variation of amino acid replacements occurring at specific structural environments that are tolerated within the predicted ΔΔG, the predicted mutation induced changes in protein thermodynamic stability in Gibbs. Both Ala1680Thr and Arg2161Trp have negative effects on protein stability, especially Ala1680Thr.

ACC1, one of the key enzymes in lipogenesis, catalyzes the conversion of acetyl-CoA into malonyl-CoA (Wakil and Abu-Elheiga, 2009). *In vivo* studies (Hasslacher et al., 1993; Baud et al., 2003; Abu-Elheiga et al., 2005) revealed the important role that ACC1 played in embryonic development. Hasslacher et al. (1993) found that residual ACC1 enzyme activity is essential for the viability of yeast. Interestingly, the fact that the embryonic lethal phenotype caused by ACC1 deficit could not be attenuated by supplementation with C12, C14, C16, C18, and C26 is interesting, which may be partly explained by the lipidomic profile of our patient-derived cells. The disruption of lipid homeostasis which caused ACACA gene mutations, was not simply due to a lack of lipids, the amount of C12 and C14 were increased and C16 was not significantly changed compared to age-matched control. It can be toxic to simply supplement with lipid compounds. In the plant model, the ACC1-null Arabidopsis was embryonic lethal which may be due to the lack of very long chain fatty acids (Baud et al., 2003). ACC1 homozygous mutant mice also suffered from embryonic lethality which stopped at 7.5 days, while heterozygous mutant mice presented with no obvious phenotypes including body weight compared to wild-type (Abu-Elheiga et al., 2005). Collectively, the partly residual enzyme activity in our patient may partly explain the escape from embryonic lethality. The absence of matched patients carrying ACC1 variants from online databases (Sobreira et al., 2015) and in our collaborators’ research may partly be due to embryonic lethality. Combined with the clues from the *in vivo* studies, screening the *ACACA* gene for recurrent spontaneous abortion may result in a “surprise.”

*In vivo* studies based on ACC1-null models revealed that the ACACA gene is essential for embryonic development, and residual enzyme activity affected the embryo survival of mutants (Hasslacher et al., 1993; Baud et al., 2003; Abu-Elheiga et al., 2005). Our *in vitro* analysis indicated that cell migratory ability in patient-derived cells was dramatically decreased by ACC1 deficit. Furthermore, the degree of migration deficit was in accordance with residual ACC1. Considering both variants were inherited and not *de novo*, the variants are potentially life-long rather than just present in the embryonic period. Cell migration is necessary throughout the embryonic development process (Kerosuo and Bronner-Fraser, 2012; Lim and Thiery, 2012; Barriga and Mayor, 2015), which includes gastrulation, somitogenesis, and neural crest migration. For example, DYNC1H1 (which encodes the heavy chain protein of the cytoplasmic dynein 1 motor protein complex) mutations cause a defect in neuronal migration causing brain development deficiencies that present as intellectual disability (Willemsen et al., 2012). Here, in our patient, the phenotype of global developmental delay especially neurological developmental delay may occur due to neural crest migration deficit. As a high ability, directional migration is required during the process of embryonic development. Of course, migration involves cellular processes including cytoskeletal reorganization, adhesion, extracellular matrix, and chemotactic signaling. The migrated deficit could associate with numerous clinical phenotypes (Landrum et al., 2020).

As outlined before, *in vitro* functional studies indicated that cell migration capacity was dramatically reduced when ACC1 became deficient. The anti-migratory action induced by ACC1 deficiency might depend on the portion of different chain length fatty acids and the portion of saturated and unsaturated fatty acids according to the lipidomic results. As reported before (Glatzel et al., 2018), an increase of desaturated fatty acids is expected to decrease the cell migratory action. As mentioned, the total amount of saturated fatty acid in PG and PC both increased in the patient-derived lymphocytes, while MUFAs presented a decrease. Interestingly, the amount of PUFA in the PC subgroup showed a dramatic increase. According to the results (Glatzel et al., 2018), MUFAs would not affect the cell migratory capacity and an increase of PUFAs would mimic the antimigratory action of ACC1 inhibition. Here, we draw the conclusion that the increase of PUFAs in the PC subgroup contributed to cell migration disability. Besides, evidence has shown that acetyl-CoA would drive mitochondrial protein acetylation (Pougovkina et al., 2014), considering an accumulation of acetyl-CoA may occur due to the ACC deficit. The overall acetylation level was detected by immunoblotting, while no difference was found between the patient cell and control (data not show).

RNAi ACC1-KD fibroblast cell lines were applied to test if the cell migration deficiency detected in patient-derived cells was induced by ACC1 deficit. The decreased ACC1 enzyme activity is in accordance with the ACC1 mRNA levels and protein levels in ACC1-KD cell lines, indicating that the methodology of ACC1 enzyme activity is promising. Glatzel et al. (2018) demonstrated that ACC1 deficit would induce cell migration deficiency by using HUVEC. Supplementation of PA in HUVEC could also partly attenuate the cell motility deficit (Supplementary Figures 3A–G). However, HUVECs are not easy to get from patients. Blood or punctured skin are often used for the functional verification of variants with unknown significance (VUS) in inherited disease patients. According to the Human Protein Atlas, ACC1 expression level in fibroblasts is almost 66.7% compared to HUVEC, however, in our data the expression level in fibroblasts and lymphocytes was even higher than HUVEC (Supplementary Figure 3H). Here, we may give some clues from the functional assays that could be applied in patients carrying VUS in the *ACACA* gene.

As lack of lipogenesis is associated with significant morbidity and mortality, early diagnosis and treatment could dramatically improve these outcomes. Fatty acid beta-oxidation would be a source of energy during long-time fasting and increase energy consumption, and FAODs would cause a deficit in energy production (Almannai et al., 2019). Recently, it was shown that the treatment for FAODs mainly focused on MCT oil, carnitine, and riboflavin supplementation, but certain types of fatty acid compensation should be forbidden according to the different types of acyl-CoA dehydrogenase deficiency. Considering ACC1 is the rate-limiting enzyme of lipogenesis, MCT oil could be a good supplementation for the patient to compensate not only for the energy deficit but also for the components of certain lipids. Our patient presented with an improvement with some of the clinical presentations. However, only one patient has been studied here, larger randomized, controlled, therapeutic trials are needed to evaluate this current understanding and further implement therapeutic strategies. Combined with our results before, ACC1 deficiency patients may benefit from the supplementation of VLCFA.

Here, we first describe a patient with *ACACA* mutations with the support of direct sequencing evidence. Functional assays were performed to investigate the pathogenesis of ACC1 deficiency and possible therapeutic chemical input. However, further conclusions about genotype-phenotype correlations and possible treatment require more clues in the future and should be carefully characterized at a clinical level.

## Conclusion

We report an individual with biallelic mutations in ACACA, presenting global development delay. *In vitro* studies revealed a disruption of lipid homeostasis in patient-derived cells, further inducing the deficit of cell motility capacity, and the deficiency could be partly attenuated by palmitate.

## Data Availability Statement

The data presented in the study are deposited in the “MetaboLights” repository, accession number “MTBLS3336.”

## Ethics Statement

The studies involving human participants were reviewed and approved by the Ethics Committee of Peking University First Hospital. Written informed consent to participate in this study was provided by the participants’ legal guardian/next of kin. Written informed consent was obtained from the individual(s), and minor(s)’ legal guardian/next of kin, for the publication of any potentially identifiable images or data included in this article.

## Author Contributions

HZ, JL, and XLo conceived and designed the experiments. XLo, XZ, HL, XB, HC, KY, and XLi performed the experiments and analyzed the data. HZ, XLo, XLu, and YY evaluated the candidate’s clinical symptoms and performed the appropriate treatment. XLo and XZ drafted the manuscript. HF, HZ, and JL proofread the manuscript. All authors read and approved the manuscript.

## Conflict of Interest

The authors declare that the research was conducted in the absence of any commercial or financial relationships that could be construed as a potential conflict of interest.

## Publisher’s Note

All claims expressed in this article are solely those of the authors and do not necessarily represent those of their affiliated organizations, or those of the publisher, the editors and the reviewers. Any product that may be evaluated in this article, or claim that may be made by its manufacturer, is not guaranteed or endorsed by the publisher.

## Footnotes

1
http://sift-dna.org

2
http://provean.jcvi.org/index.php

3
http://www.mutationtaster.org/

4
http://genetics.bwh.harvard.edu/pph2/

5
http://www.bioinfo.org.cn

6
https://www.ncbi.nlm.nih.gov/clinvar/

7
https://gnomad.broadinstitute.org

8
http://marid.bioc.cam.ac.uk/sdm2

## References

[B1] Abu-Elheiga L. Brinkley W. R. Zhong L. Chirala S. S. Woldegiorgis G. Wakil S. J. (2000). The subcellular localization of acetyl-CoA carboxylase 2. *Proc. Natl. Acad. Sci. U. S. A.* 97 1444–1449. 10677481 10.1073/pnas.97.4.1444 PMC26453

[B2] Abu-Elheiga L. Matzuk M. M. Abo-Hashema K. A. Wakil S. J. (2001). Continuous fatty acid oxidation and reduced fat storage in mice lacking acetyl-CoA carboxylase 2. *Science* 291 2613–2616. 10.1126/science.1056843 11283375

[B3] Abu-Elheiga L. Matzuk M. M. Kordari P. Oh W. Shaikenov T. Gu Z. (2005). Mutant mice lacking acetyl-CoA carboxylase 1 are embryonically lethal. *Proc. Natl. Acad. Sci. U. S. A.* 102 12011–12016. 10.1073/pnas.0505714102 16103361 PMC1189351

[B4] Adzhubei I. A. Schmidt S. Peshkin L. Ramensky V. E. Gerasimova A. Bork P. (2010). A method and server for predicting damaging missense mutations. *Nat. Methods* 7 248–249. 10.1038/nmeth0410-248 20354512 PMC2855889

[B5] Agmon E. Stockwell B. R. (2017). Lipid homeostasis and regulated cell death. *Curr. Opin. Chem. Biol.* 39 83–89. 10.1016/j.cbpa.2017.06.002 28645028 PMC5581689

[B6] Almannai M. Alfadhel M. El-Hattab A. W. (2019). Carnitine Inborn Errors of Metabolism. *Molecules* 24:3251. 10.3390/molecules24183251 31500110 PMC6766900

[B7] Bai J. Cervantes C. Liu J. He S. Zhou H. Zhang B. (2017). DsbA-L prevents obesity-induced inflammation and insulin resistance by suppressing the mtDNA release-activated cGAS-cGAMP-STING pathway. *Proc. Natl. Acad. Sci. U. S. A.* 114 12196–12201. 10.1073/pnas.1708744114 29087318 PMC5699051

[B8] Barriga E. H. Mayor R. (2015). Embryonic cell-cell adhesion: a key player in collective neural crest migration. *Curr. Top. Dev. Biol.* 112 301–323. 25733144 10.1016/bs.ctdb.2014.11.023

[B9] Baud S. Guyon V. Kronenberger J. Wuillème S. Miquel M. Caboche M. (2003). Multifunctional acetyl-CoA carboxylase 1 is essential for very long chain fatty acid elongation and embryo development in Arabidopsis. *Plant J.* 33 75–86. 10.1046/j.1365-313x.2003.016010.x 12943542

[B10] Berod L. Friedrich C. Nandan A. Freitag J. Hagemann S. Harmrolfs K. (2014). De novo fatty acid synthesis controls the fate between regulatory T and T helper 17 cells. *Nat. Med.* 20 1327–1333. 10.1038/nm.3704 25282359

[B11] Blom W. de Muinck Keizer S. M. Scholte H. R. (1981). Acetyl-CoA carboxylase deficiency: an inborn error of de novo fatty acid synthesis. *N. Engl. J. Med.* 305 465–466. 10.1056/nejm198108203050820 6114432

[B12] Choi Y. Chan A. P. (2015). PROVEAN web server: a tool to predict the functional effect of amino acid substitutions and indels. *Bioinformatics* 31 2745–2747. 10.1093/bioinformatics/btv195 25851949 PMC4528627

[B13] Clemente F. J. Cardona A. Inchley C. E. Peter B. M. Jacobs G. Pagani L. (2014). A Selective Sweep on a Deleterious Mutation in CPT1A in Arctic Populations. *Am. J. Hum. Genet.* 95 584–589. 10.1016/j.ajhg.2014.09.016 25449608 PMC4225582

[B14] Deschauer M. Wieser T. Zierz S. (2005). Muscle carnitine palmitoyltransferase II deficiency: clinical and molecular genetic features and diagnostic aspects. *Arch. Neurol.* 62 37–41. 10.1001/archneur.62.1.37 15642848

[B15] Fang H. Hu N. Zhao Q. Wang B. Zhou H. Fu Q. (2018). mtDNA Haplogroup N9a Increases the Risk of Type 2 Diabetes by Altering Mitochondrial Function and Intracellular Mitochondrial Signals. *Diabetes* 67 1441–1453. 10.2337/db17-0974 29735607

[B16] Glatzel D. K. Koeberle A. Pein H. Löser K. Stark A. Keksel N. (2018). Acetyl-CoA carboxylase 1 regulates endothelial cell migration by shifting the phospholipid composition. *J. Lipid Res.* 59 298–311. 10.1194/jlr.m080101 29208696 PMC5794424

[B17] Harwood H. J. Jr. (2004). Acetyl-CoA carboxylase inhibition for the treatment of metabolic syndrome. *Curr. Opin. Investig. Drugs* 5 283–289. 15083594

[B18] Harwood H. J. Jr. (2005). Treating the metabolic syndrome: acetyl-CoA carboxylase inhibition. *Expert Opin. Ther. Targets* 9 267–281. 10.1517/14728222.9.2.267 15934915

[B19] Hasslacher M. Ivessa A. S. Paltauf F. Kohlwein S. D. (1993). Acetyl-CoA carboxylase from yeast is an essential enzyme and is regulated by factors that control phospholipid metabolism. *J. Biol. Chem.* 268 10946–10952. 10.1016/s0021-9258(18)82077-4 8098706

[B20] Hopwood D. A. Sherman D. H. (1990). Molecular genetics of polyketides and its comparison to fatty acid biosynthesis. *Annu. Rev. Genet.* 24 37–66. 10.1146/annurev.ge.24.120190.000345 2088174

[B21] Kerosuo L. Bronner-Fraser M. (2012). What is bad in cancer is good in the embryo: importance of EMT in neural crest development. *Semin. Cell Dev. Biol.* 23 320–332. 10.1016/j.semcdb.2012.03.010 22430756 PMC3345076

[B22] Landrum M. J. Chitipiralla S. Brown G. R. Chen C. Gu B. Hart J. (2020). ClinVar: improvements to accessing data. *Nucleic Acids Res.* 48 D835–D844. 31777943 10.1093/nar/gkz972 PMC6943040

[B23] Lim J. Thiery J. P. (2012). Epithelial-mesenchymal transitions: insights from development. *Development* 139 3471–3486. 10.1242/dev.071209 22949611

[B24] Lou X. Shi H. Wen S. Li Y. Wei X. Xie J. (2018). A Novel NDUFS3 mutation in a Chinese patient with severe Leigh syndrome. *J. Hum. Genet.* 63 1269–1272. 10.1038/s10038-018-0505-0 30140060

[B25] Merritt J. L. II Norris M. Kanungo S. (2018). Fatty acid oxidation disorders. *Ann. Transl. Med.* 6:473. 10.21037/atm.2018.10.57 PMC6331364 30740404

[B26] Milgraum L. Z. Witters L. A. Pasternack G. R. Kuhajda F. P. (1997). Enzymes of the fatty acid synthesis pathway are highly expressed in in situ breast carcinoma. *Clin. Cancer Res.* 3 2115–2120. 9815604

[B27] Miller M. J. Burrage L. C. Gibson J. B. Strenk M. E. Lose E. J. Bick D. P. (2015). Recurrent ACADVL molecular findings in individuals with a positive newborn screen for very long chain acyl-coA dehydrogenase (VLCAD) deficiency in the United States. *Mol. Genet. Metab.* 116 139–145. 10.1016/j.ymgme.2015.08.011 26385305 PMC4790081

[B28] Pandurangan A. P. Blundell T. L. (2020). Prediction of impacts of mutations on protein structure and interactions: SDM, a statistical approach, and mCSM, using machine learning. *Protein Sci.* 29 247–257. 10.1002/pro.3774 31693276 PMC6933854

[B29] Pohle T. Brändlein S. Ruoff N. Müller-Hermelink H. K. Vollmers H. P. (2004). Lipoptosis: tumor-specific cell death by antibody-induced intracellular lipid accumulation. *Cancer Res.* 64 3900–3906. 10.1158/0008-5472.can-03-3149 15173000

[B30] Pougovkina O. te Brinke H. Ofman R. van Cruchten AG Kulik W. Wanders R. J. (2014). Mitochondrial protein acetylation is driven by acetyl-CoA from fatty acid oxidation. *Hum. Mol. Genet.* 23 3513–3522. 10.1093/hmg/ddu059 24516071

[B31] Richards S. Aziz N. Bale S. Bick D. Das S. Gastier-Foster J. (2015). Standards and guidelines for the interpretation of sequence variants: a joint consensus recommendation of the American College of Medical Genetics and Genomics and the Association for Molecular Pathology. *Genet. Med.* 17 405–424. 10.1038/gim.2015.30 25741868 PMC4544753

[B32] Rios Garcia M. Steinbauer B. Srivastava K. Singhal M. Mattijssen F. Maida A. (2017). Acetyl-CoA Carboxylase 1-Dependent Protein Acetylation Controls Breast Cancer Metastasis and Recurrence. *Cell Metab.* 26 842–855.e5. 29056512 10.1016/j.cmet.2017.09.018

[B33] Schwarz J. M. Cooper D. N. Schuelke M. Seelow D. (2014). MutationTaster2: mutation prediction for the deep-sequencing age. *Nat. Methods* 11 361–362. 10.1038/nmeth.2890 24681721

[B34] Sherry S. T. Ward M. H. Kholodov M. Baker J. Phan L. Smigielski E. M. (2001). dbSNP: the NCBI database of genetic variation. *Nucleic Acids Res.* 29 308–311. 10.1093/nar/29.1.308 11125122 PMC29783

[B35] Sims D. Sudbery I. Ilott N. E. Heger A. Ponting C. P. (2014). Sequencing depth and coverage: key considerations in genomic analyses. *Nat. Rev. Genet.* 15 121–132. 10.1038/nrg3642 24434847

[B36] Sobreira N. Schiettecatte F. Valle D. Hamosh A. (2015). GeneMatcher: a matching tool for connecting investigators with an interest in the same gene. *Hum. Mutat.* 36 928–930. 10.1002/humu.22844 26220891 PMC4833888

[B37] Stiede K. Miao W. Blanchette H. S. Beysen C. Harriman G. Harwood H. J. Jr. (2017). Acetyl-coenzyme A carboxylase inhibition reduces de novo lipogenesis in overweight male subjects: a randomized, double-blind, crossover study. *Hepatology* 66 324–334. 10.1002/hep.29246 28470676 PMC5599970

[B38] Sumiya N. Kawase Y. Hayakawa J. Matsuda M. Nakamura M. Era A. (2015). Expression of Cyanobacterial Acyl-ACP Reductase Elevates the Triacylglycerol Level in the Red Alga *Cyanidioschyzon merolae*. *Plant Cell Physiol.* 56 1962–1980. 10.1093/pcp/pcv120 26272551

[B39] Thampy K. G. (1989). Formation of malonyl coenzyme A in rat heart. Identification and purification of an isozyme of A carboxylase from rat heart. *J. Biol. Chem.* 264 17631–17634. 10.1016/s0021-9258(19)84614-8 2572585

[B40] Vaser R. Adusumalli S. Leng S. N. Sikic M. Ng P. C. (2016). SIFT missense predictions for genomes. *Nat. Protoc.* 11 1–9. 10.1038/nprot.2015.123 26633127

[B41] Wakil S. J. Abu-Elheiga L. A. (2009). Fatty acid metabolism: target for metabolic syndrome. *J. Lipid Res.* 50 S138–S143. 19047759 10.1194/jlr.R800079-JLR200 PMC2674721

[B42] Willemsen M. H. Vissers L. E. Willemsen M. A. van Bon B. W. Kroes T. de Ligt J. (2012). Mutations in DYNC1H1 cause severe intellectual disability with neuronal migration defects. *J. Med. Genet.* 49 179–183. 10.1136/jmedgenet-2011-100542 22368300

[B43] Willis L. B. Omar W. S. W. Sambanthamurthi R. Sinskey A. J. J. (2008). Non-radioactive assay for acetyl-CoA carboxylase activity. *J. Oil Palm Res.* 2 30–36.

[B44] Worth C. L. Preissner R. Blundell T. L. (2011). SDM—a server for predicting effects of mutations on protein stability and malfunction. *Nucleic Acids Res.* 39 W215–W222. 21593128 10.1093/nar/gkr363 PMC3125769

[B45] Zhu J. Thompson C. B. (2019). Metabolic regulation of cell growth and proliferation. *Nat. Rev. Mol. Cell Biol.* 20 436–450. 10.1038/s41580-019-0123-5 30976106 PMC6592760

