# Supplementary material for: Biallelic Mutations in ACACA Cause a Disruption in Lipid Homeostasis That Is Associated With Global Developmental Delay, Microcephaly, and Dysmorphic Facial Features
Source: Front Cell Dev Biol. 2021 Sep 6;9:618492. doi: 10.3389/fcell.2021.618492 (PMC8450402; doi:10.3389/fcell.2021.618492)
Supplement: Supplementary file 1 [file Data_Sheet_1.docx]

**Supplemental Data**

Biallelic mutations in *ACACA* cause a disruption in lipid homeostasis that is associated with global developmental delay, microcephaly, and dysmorphic facial features

A B


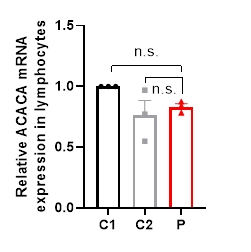
C D


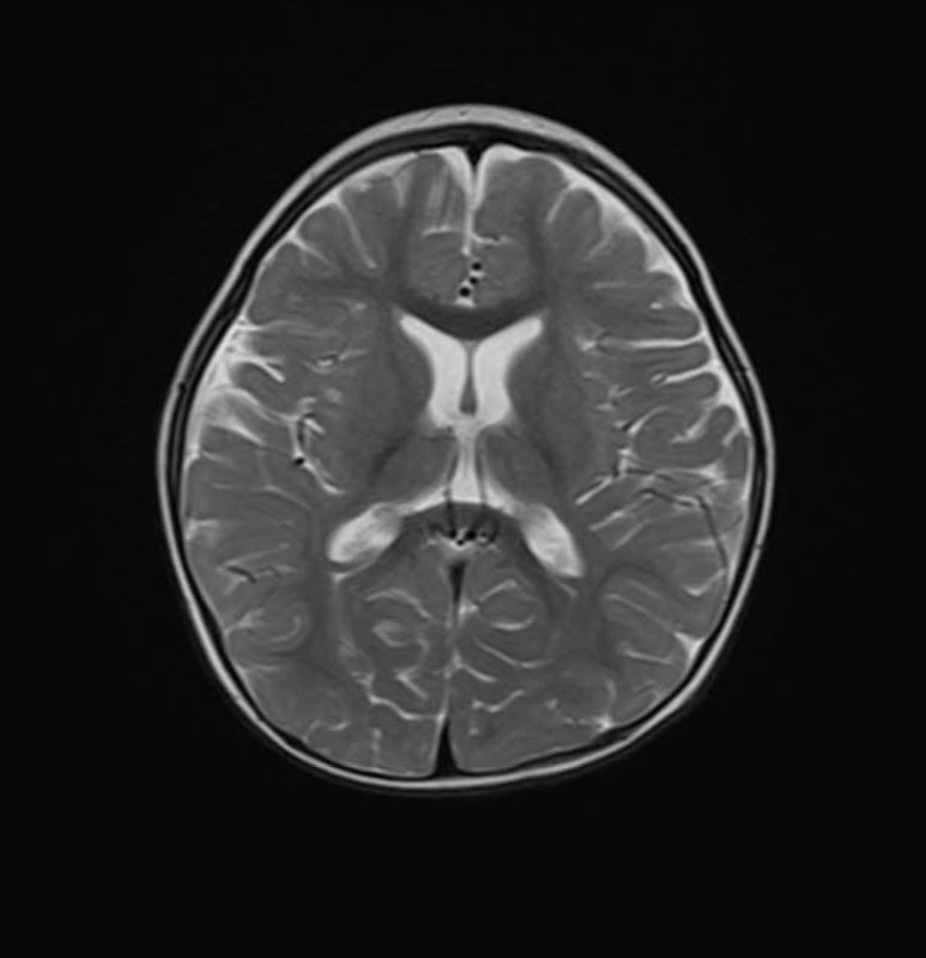

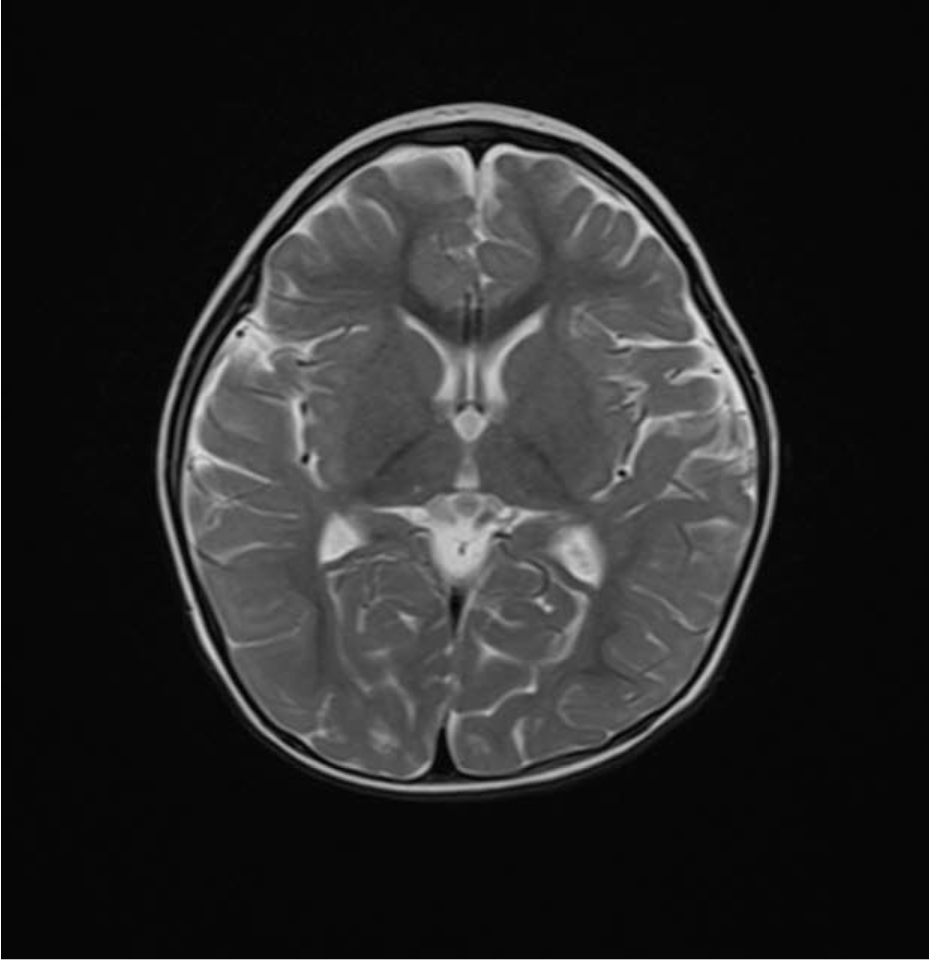


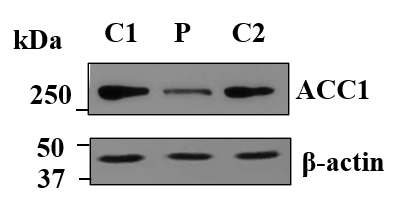

**Figure S1** **(A)** Brain MRI of patient, the arrows indicate the aberrant of the bilateral cerebral sulci; **(B)** *ACACA* mRNA expression in patient- and controls-derived lymphocytes. **(C)** ACC1 protein level in cytoplasm. **(D)** ACC1 enzyme activity in patient-derived lymphocytes normalized with the ACC1 protein level. N.s., no statistical significance. *p<0.05. One-way *ANOVA* applied in fig b, and d.


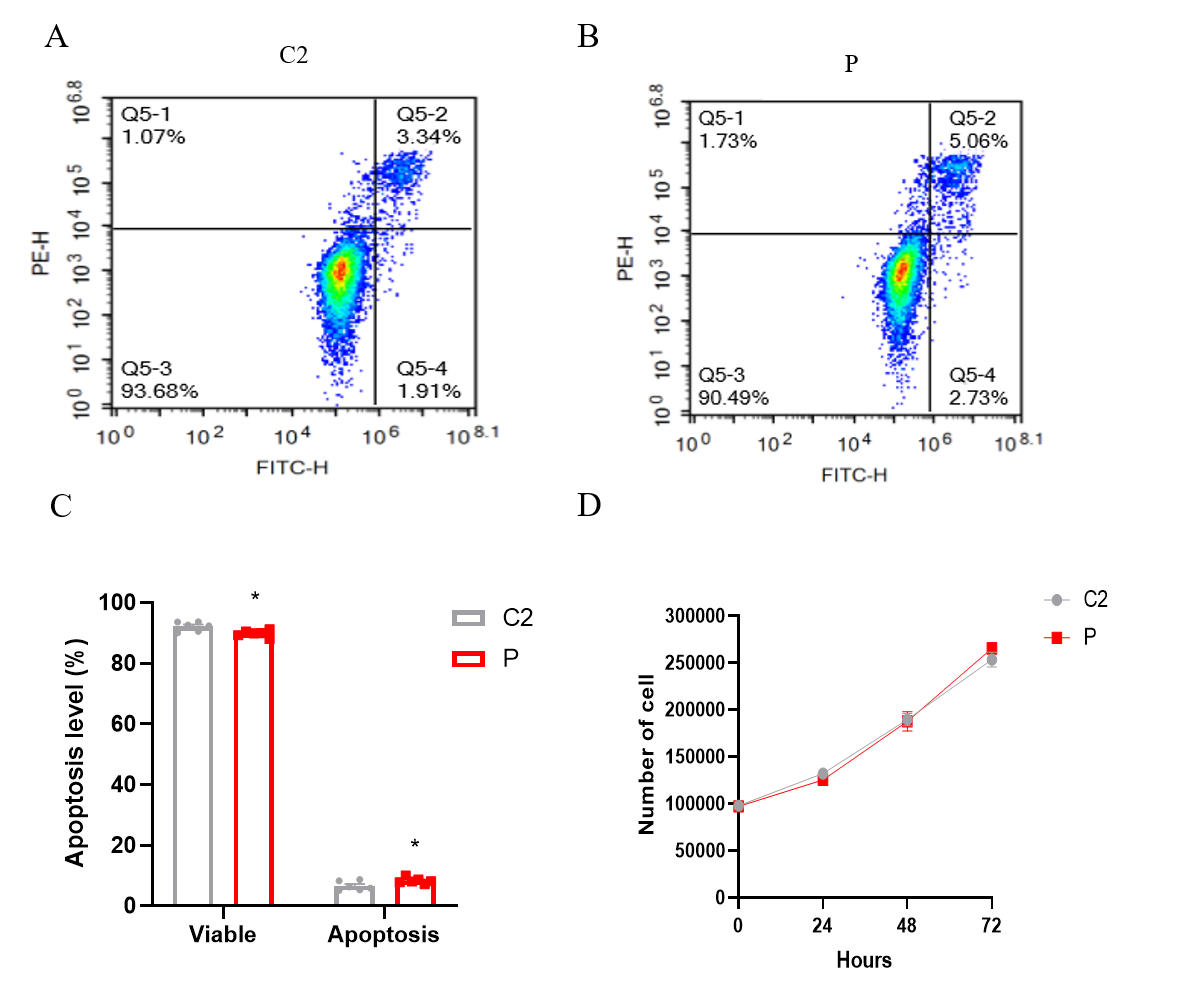


**Figure S2: (A) (B)** FITC and PI staining lymphocytes were detected by flow cytometer, and **(C)** the percentage of viable and the apoptotic cells. **(D)** Cell number of patient- and age-matched control-derived lymphocytes were detected in different time points. *p<0.05. Independent *t*-test in fig c-d.


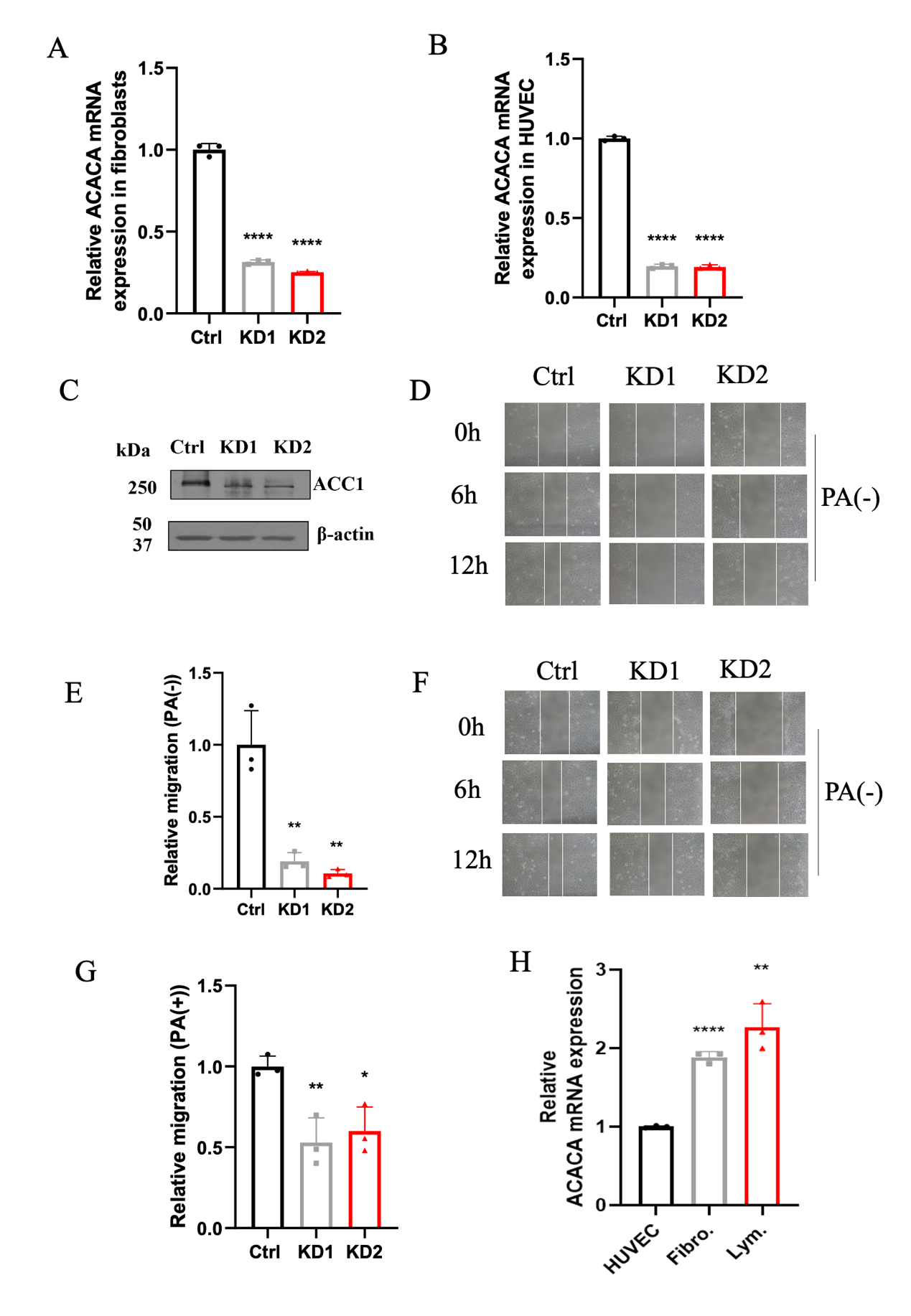


**Figure S3** **(A)** Relative *ACACA* mRNA expression in fibroblast cell lines. **(B)** Relative *ACACA* mRNA expression in HUVEC cell lines. **(C)** ACC1 protein expression in ACC1-KD HUVEC cell lines. **(D) (E)** The wound healing assay of HUVEC cell lines and the quantification of wound healing capacity. **(F) (G)** The wound healing assay of HUVEC cell lines with the supplement of PA and the quantitative analysis. **(H)** The expression of *ACACA* mRNA level in fibroblasts and lymphocytes relative to HUVEC cell line. *p<0.05, **p<0.01, ***p<0.001, ****p<0.0001. Independent *t*-test in fig a, b, e, g, and h.

**Table S1. Summary of clinical and laboratory data in patient before or after treatment.**

|  | | | Age (month) | | | |
| --- | --- | --- | --- | --- | --- | --- |
| **Clinical feature** | |  | 0 | 25 (Before treatment) | 44(After treatment) | 51(After treatment) |
| Height | |  | 50cm | 83cm (-2SD<<-1SD) | 96.3cm (-2SD<<-1SD) | NS |
| Weight | |  | 3.3kg | 11.5kg (-2SD<<-1SD) | 14kg (-2SD<<-1SD) | NS |
| Head circumference | |  | NS | 43.5cm (<-3SD) | 45.8cm (<-3SD<<-2SD) | NS |
| **GMDS** | |  | Raw score  (Equivalent age/Actual Age, %) | | | |
|  | Gross motor quotient | | NS | 11.3 (11/25, 44%) | 20 (19.5/44, 44%) | 20(20/51, 39%) |
|  | Personal social emotional | | NS | 9.9 (9/25, 36%) | 20 (18/44, 41%) | 22(19.5/51, 37%) |
|  | Language and communication | | NS | 7.5 (7.5 /25, 30%) | 19 (20/44, 45%) | 24(24.5, 48%) |
|  | Eye and hand coordination | | NS | 6.4 (6.5 /25, 20%) | 17 (18/44, 41%) | 18(19/51, 37%) |
|  | Vision | | NS | 8.2 (8.5/25, 34%) | 21(21.5/44, 49%) | 23(23.5, 46%) |
| **Neurological Findings** | |  |  |  |  |  |
| Muscle tone | |  | NS | Decreased | NS | NS |
| Muscle strength | |  | NS | IV | NS | NS |
| Mental status | |  | NS | Sluggish, language disorder, fatigue. | Simple communication, poor comprehension and improved fatigue. | Simple communication and comprehension, basic physical strength, imitate simple motion. |

Abbreviation: GMDS, The Griffith Mental Development Scales; NS, No assessment; SD: Standard deviation.

**Table S2.** **Primers used for this paper**

| Name | Use | Primers |
| --- | --- | --- |
| ACACA-1 | PCR for c.4858G>A | F-5-AAGTGCCTGACTTTTGGCAT-3 |
|  |  | R-5-TGACCACCTCACAGAAAGCTG-3 |
| ACACA-2 | PCR for c.6481C>T | F-5-AGGGAAAAGAGGGCATGTGG-3 |
|  |  | R-5-ACATGTGGGCCTCTGACAAG -3 |
| ACACA | qPCR for ACACA | F-5-AGATGTTTCGGCAGTCCCTG-3 |
|  |  | R-5-ATGTGGACCAGCTGACCTTG-3 |
| siRNA | Knockdown of *ACACA* expression | 5- GCAGAAACUCAUCCUAUCATT -3(KD1) |
|  |  | 5-GCAGAAACUCAUCCUAUCATT-3 (KD2) |
| β-actin | qPCR for β-actin | F-5-GACCTGTACGCCAACACAGT-3 |
|  |  | R-5-AGTACTTGCGCTCAGGAGGA-3 |
